# Beyond N-Cadherin, Relevance of Cadherins 5, 6 and 17 in Cancer Progression and Metastasis

**DOI:** 10.3390/ijms20133373

**Published:** 2019-07-09

**Authors:** J. Ignacio Casal, Rubén A. Bartolomé

**Affiliations:** Department of Molecular Biomedicine, Centro de Investigaciones Biológicas (CIB)-Consejo Superior de Investigaciones Científicas (CSIC), Ramiro de Maeztu 9, 28039 Madrid, Spain

**Keywords:** cadherin 17 (CDH17), VE-cadherin, cadherin 6 (CDH6), N-cadherin, α2β1 integrin, RGD motif, metastasis, therapeutic antibodies

## Abstract

Cell-cell adhesion molecules (cadherins) and cell-extracellular matrix adhesion proteins (integrins) play a critical role in the regulation of cancer invasion and metastasis. Although significant progress has been made in the characterization of multiple members of the cadherin superfamily, most of the published work continues to focus in the switch E-/N-cadherin and its role in the epithelial–mesenchymal transition. Here, we will discuss the structural and functional properties of a subset of cadherins (cadherin 17, cadherin 5 and cadherin 6) that have an RGD motif in the extracellular domains. This RGD motif is critical for the interaction with α2β1 integrin and posterior integrin pathway activation in cancer metastatic cells. However, other signaling pathways seem to be affected by RGD cadherin interactions, as will be discussed. The range of solid tumors with overexpression or “de novo” expression of one or more of these three cadherins is very wide (gastrointestinal, gynaecological and melanoma, among others), underscoring the relevance of these cadherins in cancer metastasis. Finally, we will discuss different evidences that support the therapeutic use of these cadherins by blocking their capacity to work as integrin ligands in order to develop new cures for metastatic patients.

## 1. Introduction

Metastasis initiation is enabled by cellular plasticity, including the mesenchymal-to-epithelial transition (MET) and the gain of stem-cell-like properties by cells in the metastatic organ site [1]. Organ-specific metastasis depends on different molecules, including growth factors, receptors, proteases, chemokines and cellular adhesion molecules, such as cadherins and integrins [2,3]. Cadherins are a major class of cell–cell adhesion molecules that regulate the adhesion and migration of cells in a calcium-dependent manner [4,5]. Cell–cell adhesion regulates cell polarity, cell differentiation and participates in the establishment of tissue homeostasis. Epithelial cadherin (E-cadherin, CDH1) is considered the prototype cadherin due to its early identification and exhaustive characterization [6]. It has been involved in stem cell maintenance and differentiation [7]. E-cadherin is considered to be a tumor suppressor involved in the epithelial–mesenchymal transition (EMT) [8]. During the EMT process, repression of E-cadherin by the transcription factor Snail and others (Slug, Zeb1, Zeb2) provokes the loss of the epithelial properties and a higher migration and invasion capacity of the cancer cells. EMT is characterized by the so-called “cadherin switch” between E-cadherin and the less adhesive neural cadherin (N-cadherin). Many cancer-related studies have mainly focused on this cadherin switch from E-cadherin, which is highly expressed in epithelial cells, to the N-cadherin, which is preferentially expressed in cells with a more mesenchymal phenotype [9]. Cadherin composition fluctuates during tumor progression and metastasis as a response to the different microenvironments. Loss of E-cadherin weakens the adherens junctions involved in cell–cell adhesion and facilitates the release of the cells from the primary tumors. N-cadherin was reported to promote aggregation and collective cell migration that facilitates invasion and metastasis [10,11,12]. Indeed, there exists a vast literature describing the increase of N-cadherin expression in cancer metastasis (see [13] for a recent review).

Despite the increasing number of publications describing the association of other cadherins which are different to E- and N-cadherin in terms of cancer development and progression, the characterization of these alternative cadherins remains poorly investigated. In this review, we will discuss the structural and functional properties of cadherin 5 (CDH5), cadherin 6 (CDH6) and cadherin 17 (CDH17), as well as their pathological implications in cancer progression and metastasis. These cadherins have, in common, the presence of RGD motifs within their sequence. Although another two cadherins, CDH16 and CDH20, contain RGD motifs, they will not be reviewed here as their relationship with cancer metastasis is not well established. In addition, we will discuss the implication of these three RGD cadherins in cancer signalling pathways and how this signalling might be regulated by the interaction network of each cadherin. Finally, we will address their emerging value as therapeutic targets for the treatment of cancer metastasis.

## 2. RGD Cadherins Structure and Function

### 2.1. Cadherin 17

CDH17 (also known as liver–intestine cadherin or LI-cadherin), together with CDH16, belongs to the 7D cadherin family, characterized by having seven extracellular (EC) domains [14]. CDH17 has been well characterized at the structural level in the last two decades. EC domains 1–2 have originated by a duplication of the first two domains of a 5-repeat cadherin [15]. The conserved tryptophan at position 2 in the EC1 of classical cadherins has been replaced by a phenylalanine in CDH17 [16]. Both 7D cadherins contain an RGD motif, which is located in the EC6 domain in CDH17. Another relevant structural difference in 7D cadherins is the absence of a large cytoplasmic domain when compared to classical or type II cadherins. CDH17 cytoplasmic domain is very short and it has been commonly assumed that it cannot interact with the catenin proteins to bind to the actin cytoskeleton. According to this hypothesis, cell signalling would require cooperation with other molecules. However, our group recently described the association of CDH17 with β-catenin and p120-catenin using an immunoprecipitation approach [17]. Indeed, the interaction with p120-catenin might use two dileucine motifs (LL) which are present in CDH17 positions 792–793 and 796–797 just at the end of the transmembrane region. The dileucine motif is the core p120-catenin binding site used for E-cadherin endocytosis [18]. We can hypothesize that the interaction with β-catenin might use some other residues of the short cytoplasmic tail. Alternatively, we cannot discard that the interaction with the β-catenin occurs indirectly through interactions with other catenins and/or proteins.

EC domains of cadherins are usually cleaved near the plasma membrane by different proteases, including matrix metalloproteinases (MMPs) (MMP3, MMP6, MMP9, MT1-MMP (MMP14) or Disintegrin and Metalloproteinase Domain-Containing Protein 10 (ADAM10)) and kallikrein 6 in the case of E-cadherin [4]. These fragments may block the homotypic binding of cadherins to facilitate cell migration and invasion [19]. A similar shedding has been observed for CDH17 [20], although the protease(s) involved in this shedding have not been characterized yet. These soluble CDH17 fragments might be relevant for “trans” interactions between cadherin subunits or between cadherin-integrin subunits, as we will discuss later.

Regarding phylogenetic analysis, CDH17 is well preserved in all mammalians, even an ancestral *CDH17* gene was identified in zebrafish kidney development that can be considered the precursor of the mammalian genes *CDH17* and *CDH16* [21]. In physiological conditions, CDH17 is only expressed in the small and large intestine in humans and mice, but not in the upper gastric tract or the liver [22]. In mice, CDH17 expression starts at day 12.5 matching the formation of the intestinal villi and it was always co-expressed with E-cadherin in polarized cells [23]. CDH17 is located exclusively on the basolateral membrane of hepatocytes, enterocytes and goblet cells but not on the apical plasma membrane.

CDH17 is mainly a Ca^2+^-dependent cell–cell adhesion protein. These cell–cell adhesion properties induce cell clustering at a similar level to classical cadherin [23]. Interaction between CDH17 molecules occur in a trans mode and are preferentially homophilic, although some heterotypic interactions with other cadherins might be possible. Moreover, the homotypic interaction of CDH17 displays higher affinity than the corresponding interactions of classical cadherins. This property of CDH17 might be relevant for the formation of cell micro-aggregates to facilitate the formation of micrometastasis. The homotypic interaction of CDH17 is only possible in cholesterol-rich domains (“lipid rafts”) and is dependent of cholesterol levels [24]. The precise biological function of CDH17 is still a matter of debate. CDH17 has been involved in water resorption under various osmotic conditions by adjusting the intercellular cleft [25]. Therefore, CDH17 would participate in the regulation of water transport through epithelia. Water transport might be related to and/or regulated by the Ca^2+^ concentration, which is necessary to retain the CDH17-mediated adhesion. It has been published that the removal of Ca^2+^ increases the diffusion coefficient of the CDH17 in plasma membrane [26], making Ca^2+^ levels critical for cadherin trans-interactions. This CDH17 ability that combines the clustering of the EC domains in combination with high mobility might facilitate the formation of highly dynamic adhesive contacts. In summary, CDH17 cell–cell-mediated adhesion is highly dependent of Ca^2+^ concentration, which in turn, depends on the overall electrolyte concentration and the water resorption processes. It is interesting to note that Ca^2+^ alterations have been considered relevant in the hallmarks of cancer progression [27]. Although there are very few data about the relationship between CDH17 expression and proteins involved in the regulation of Ca^2+^ levels, it is noteworthy that a number of alterations in these proteins have been routinely observed in colorectal cancer metastasis. These correlations deserve further study.

### 2.2. VE-Cadherin

CDH5, also known as vascular–endothelial (VE)-cadherin, belongs to the type II cadherin family. As for other members of the family, its structure consists of five EC domains, a single-pass transmembrane domain and the cytoplasmic domain [28]. Cell–cell adhesion is established by homotypic interactions between the five EC domains that are stabilized by binding of Ca^2+^ ions to the cadherin repeats [29]. Antibodies against domains EC1 and EC4 have demonstrated a role for these regions in cell–cell adhesion and endothelial integrity [30]. RGD motifs are located in EC2 and EC3, which is different from the adhesion motifs. In a similar way to CDH17, VE-cadherin RGD motifs are present in many mammals and birds, but not in rodents or dogs [31]. The cytoplasmic tail of VE-cadherin interacts with p120-catenin, β-catenin and plakoglobin [32]. These catenins form the structural bridges with the actin cytoskeleton to enhance adhesion. These interactions are variable: in recently formed vessels; cell to cell contacts are less stable and more prone to migration and proliferation. In contrast, mature endothelial cells are more stable in order to maintain vascular structure, inhibiting cellular expansion and migration. The VE-cadherin signaling capacity in endothelial cells has been extensively reviewed (i.e., [32]). Soluble VE-cadherin fragments have been found in the blood of patients with systemic inflammation and sepsis and were involved in the endothelial barrier breakdown [33]. As described for E-cadherin, the shedding of VE-cadherin EC domains involves ADAM10 and appears to be of clinical relevance in cardiovascular diseases. Its role in cancer progression remains to be clarified.

VE-cadherin activity is modulated by phosphorylation of the Tyr residues of the cytoplasmic domain by phosphatases and kinases, which regulate signaling and junctional permeability. Three Tyr residues (Y645, Y658 and Y685) in the cytoplasmic tail are targets of different kinases (i.e., Src or C-terminal Src kinase (Csk)) [34]. Whereas Src-mediated phosphorylation promotes increased permeability, dephosphorylation by protein tyrosine phosphatases induces junctional strength. Phosphorylation of Y645 by Csk may lead to the inactivation of Src activity and, then, that of focal adhesion kinase (FAK) at adherens junctions leading to β-catenin dissociation from VE-cadherin in angiogenesis [35]. The vascular endothelial growth factor (VEGF) family is the main factor for angiogenesis regulation. VEGF may modulate the activity of the p120-catenin and β-catenin through the VEGFR2 to induce a reduced presence of VE-cadherin in the adherens junctions. Many other signaling events are modulated by VE-cadherin in endothelial cells (for a complete review see [36]). VE-cadherin is selectively expressed at the adherens junctions of the endothelial cells and, therefore, an overwhelming percentage of studies about its function are related with cardiovascular research. VE-cadherin studies in cancer progression and metastasis are much more limited and were mainly restricted to its function in vasculogenic mimicry until recently. New findings in TGFβ activation and integrin interactions demonstrated new roles for this cadherin in metastatic progression (see Section 3.2 and Section 5).

### 2.3. Cadherin 6

CDH6, also known as fetal kidney cadherin or K-cadherin, is involved in the morphogenesis and development of the embryonic kidney, where it drives the mesenchymal–epithelial differentiation that is necessary for kidney morphogenesis [37,38]. However, in the adult kidney, CDH6 expression is very weak or negligible [39,40]. CDH6 is a type II cadherin (like VE-cadherin), which has not been extensively investigated, and is characterized as containing 5 EC domains and a large cytoplasmic domain for the interaction with catenin molecules. CDH6 contains the RGD motif at the EC1 domain and the His-Ala-Val (HAV) motif for stabilization and clustering of adjacent monomers at the EC5. In contrast, human CDH17 and CDH5 do not contain HAV motifs. β-catenin binds directly to the cytoplasmic tail of cadherins and to α-catenin to regulate the actin cytoskeleton. Given the central role of β-catenin in the Wnt signaling pathway, a link between β-catenin signaling and cell–cell adhesion mediated by CDH6 has been suggested [41]. In Madin–Darby canine kidney (MDCK) cells, loss of CDH6 in the presence of active β-catenin led to more flat and less compacted colonies than control cells [41]. Therefore, CDH6-low cells tend to be more spindly, fibroblast-like and more migratory and invasive [42]. This role seems to agree with the promigratory/proinvasive properties of CDH17 low cells. CDH6 is also expressed in platelets, having a functional role in platelet aggregation and thrombus formation. This function is mediated by the binding of the RGD motif to αIIbβ3 integrin, which undergoes a conformational change and binds fibrinogen for the cross-linking of platelets [43]. From the very beginning, CDH6 was associated with high expression in renal carcinomas [44,45]. However, beyond its role in morphogenesis, platelets and cancer progression (see Section 3.3), little is known about the biological functions of CDH6.

## 3. Relevance of CDH17, CDH5 and CDH6 in Cancer Progression and Metastasis

### 3.1. Cadherin 17 in Gastrointestinal Cancers and Other Tumors

Despite some initial conflicting results, high CDH17 expression is clearly associated with metastatic progression in different neoplasias, mainly of gastrointestinal origin. Most of the previous conflicting results were due to the unknown association of CDH17 expression with the differentiation status of the tumor. Whereas well-differentiated tumors express high amounts of CDH17 in late stages, poorly-differentiated tumors do not express CDH17. High expression of CDH17 in cancer cells correlates in general with a high expression of the intestine-specific transcription factor CDX2, which regulates CDH17 expression [45] and plays a key role in the intestinal epithelial development and differentiation [46]. Therefore, some initial studies restricted to aggressive and poorly-differentiated colorectal and pancreatic carcinomas observed negligible amounts of CDH17 and presumed that CDH17 was generally reduced in advanced colorectal and pancreatic cancer. The use of a larger series of samples, including well-differentiated tumors has clarified this issue.

CDH17 is highly expressed in gastric cancer [47,48], pancreatic cancer [49,50], particularly in the exocrine-like subtype of pancreatic ductal adenocarcinoma [51], Barrett´s esophagus and associated carcinoma [52], neuroendocrine tumors [53,54], colorectal cancer [45,50,55,56] and hepatocellular carcinoma [57,58,59]. In many of these cancers, CDH17 expression is associated with tumor stage or poor survival of patients [37,38]. Indeed, CDH17 is considered a sensitive marker for the detection of metastasis of unknown primary origin and for their subsequent assignation to the gastrointestinal tract [60]. Regarding other tumors, up-regulation of CDH17 was observed in advanced stages of epithelial ovarian cancer and associated with poor prognosis [61]. CDH17 was also associated with mucinous epithelial ovarian cancer [62]. Indeed, high expression of CDH17 in this subtype of ovarian cancer can be readily observed in the Human Protein Atlas [63]. In addition, CDH17 has been used to distinguish primary adenocarcinomas of the bladder from urothelial carcinoma with glandular differentiation [64]. All these results confirm the increased relevance of CDH17 in the metastatic progression of a wide range of solid tumors.

**Figure 1:**
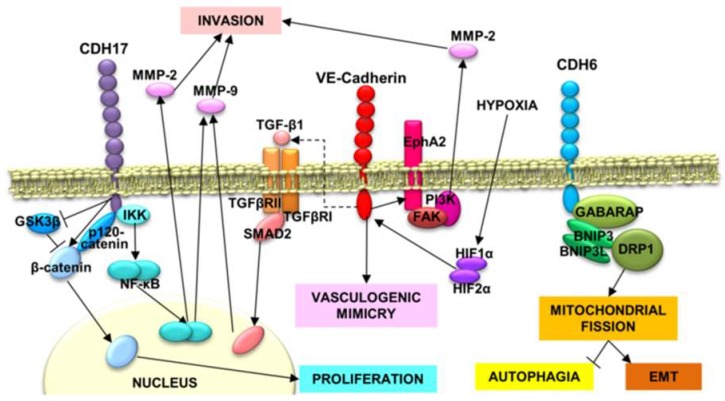
Cancer-related signaling pathways involving RGD cadherins. CDH17-triggered phosphorylation of IκB Kinase (IKK) causes NF-κB release and translocation to the nucleus, where it promotes the expression of MMP-2 and MMP-9, allowing a more efficient cell invasion of the extracellular matrix. In addition, CDH17 downregulates GSK3β activation, promoting the accumulation of β-catenin and its nuclear translocation to induce the expression of cyclins and other proteins involved in proliferation. Vascular–endothelial (VE)-cadherin expression is modulated by hypoxia-induced factors, HIF1α and HIF2α. VE-cadherin, in addition to induce vasculogenic mimicry and promotes EphA2 stabilization in cell membrane, which in turn, activates FAK and PI3K. PI3K increases the activation of MMP-2, favoring the invasion. VE-cadherin also induces the expression of TGF-β1, which induces Mothers Against Decapentaplegic Homolog 2 (SMAD2) phosphorylation and nuclear translocation to promote MMP-9 expression and, subsequently, cell invasion. CDH6, next to GABARAP, Dynamin-Related protein 1(DRP1), BNIP3 and BINP3L, promote mitochondrial fission through DRP1, which impedes autophagia and promotes the epithelial–mesenchymal transition.

Regarding gene expression regulation, an analysis of the microRNA (miRNA) expression in the highly metastatic colorectal cancer cell line KM12SM, indicated that three miRNAs (miR-424-3p, -503, and -1292), overexpressed in the metastatic cells, caused an increased expression of CDH17 [65]. This report concluded that multiple miRNAs regulate the expression of the same set of proteins, which might be necessary for more efficient control of the metastatic process. Therefore, CDH17 expression seems to be regulated in a coordinated way by multiple miRNAs. Regarding signaling, CDH17 has been implicated in different signaling pathways. In hepatocarcinoma, loss of CDH17 expression was associated with inactivation of the Wnt signaling pathway and a significant reduction of total and nuclear β-catenin and the phosphorylated form of Glycogen Synthase Kinase 3 Beta (GSK-3β) [57] (Figure 1). CDH17 has also been reported to alter MMP9 and MMP2 expression via activation of the NF-kB signaling pathway in gastric cancer [66,67] (Figure 1). In colorectal cancer, we have confirmed the interaction of CDH17 with α-, β-, and p120-catenins [17]. In addition, we have observed a large effect of CDH17 on cell adhesion and proliferation in metastatic colorectal cancer cell lines [17]. This impact was mediated by the binding of CDH17 to the α2β1 integrin and the subsequent activation of the integrin signaling pathway and increased binding to collagen IV [20]. This binding was mediated by an RGD motif present in the CDH17 in humans and other mammalians (see Section 5). Therefore, the RGD motif in cadherins seems to work as a switch that regulates integrin activation in different types of metastatic cells. The observed increase of α2 integrin expression during liver metastasis suggests that the co-expression of CDH17 and the α2 integrin is necessary for the pro-metastatic activity [68].

### 3.2. VE-Cadherin in Breast Cancer, Melanoma and Other Tumors

VE-cadherin overexpression has been reported in a number of neoplasias. In a pioneer work, VE-cadherin was associated with vasculogenic mimicry (the ability of cancer cells to form novel blood-vessel-like structures) in uveal melanoma [69,70]. This capacity was further confirmed in glioblastoma stem-like cells [71] and many other tumors [72]. Vasculogenic mimicry constitutes an alternative way to secure the blood supply to tumors and makes the use of antiangiogenic drugs in cancer more inefficient. Vasculogenic mimicry is a key process in pediatric cancers as astrocytomas [73] and rhabdomyosarcomas [74] and is correlated with poor survival. In other childhood cancers, VE-cadherin is overexpressed in Ewing´s sarcoma [75] and is a stem cell marker of Philadelphia positive (Ph+) cells, including Ph+ acute lymphoblastic leukemia (ALL) and chronic myeloid leukemia blast crisis cells, where CDH5 contributes to cell survival [76]. Expression of VE-cadherin and Platelet and Endothelial Cell Adhesion Molecule 1 (PECAM-1) by ALL cells enhanced adhesion and migration across brain microvascular endothelial cell monolayers [77]. Indeed, VE-cadherin has been suggested as a potential target for the treatment of Ph+ leukemias [78]. VE-cadherin is also overexpressed in lung cancer [79] and highly aggressive cutaneous melanomas [80]. Recently, VE-cadherin has been postulated as a biomarker for metastatic breast cancer [81], particularly in estrogen-receptor-positive breast cancers with vascular invasion, where its expression correlates with metastasis [82]. CDH5 mRNA was also found up-regulated in pancreatic neuroendocrine tumors in sporadic and Von-Hippel Lindau patients [83]. High CDH5 expression correlates with tumor progression, metastasis and poor survival in patients with differentiated-type gastric cancer [84]. In esophageal cancer tissues, CDH5 mRNA expression correlated with the expression of VEGF molecules [85]. VEGF expression is higher in differentiated tubular adenocarcinomas than in signet-ring cell carcinoma.

An initial characterization of the VE-cadherin role in cancer cell signaling was made in the mouse mammary epithelial cell line EpH4 transformed by the v-Ha-Ras oncogene, where there was a significant increase of VE-cadherin expression, accompanied by a down-regulation of E-cadherin [86]. This effect was associated with EMT, as treatment with Transforming Growth Factor beta (TGF-β) of the same parental cells caused similar alterations in phenotype and protein expression in the long-term (Figure 1). However, the authors clearly established that VE-cadherin was not a target directly regulated by TGF-β. Regarding vasculogenic mimicry, CDH5 regulates EPH Receptor A2 (EphA2) expression in melanoma cells [87] and, conversely, CDH5 expression was significantly decreased after knockdown of hypoxia-inducible factors (HIF1α or HIF2α) in glioblastoma [88] (Figure 1). Therefore, hypoxic conditions would induce CDH5 participation in vasculogenic mimicry. In addition, we reported the interaction of CDH5 with α2β1 integrin in melanoma and breast cancer cells to activate the integrin signaling pathway, promoting migration and invasion and, consequently, metastasis in a similar way to CDH17 [31]. In summary, several mechanisms have been proposed to explain the VE-cadherin role in vasculogenic mimicry, cancer progression and metastasis. Further work is necessary to interconnect the different mechanisms and to clarify if they are cell-context specific.

### 3.3. Cadherin 6 Expression in Different Tumors

According to its initial location, CDH6 was mainly implicated in renal carcinomas, where it correlates with lymph node invasion and metastasis [43,44,89]. In addition, CDH6 expression has been observed in ovarian carcinoma [90,91], thyroid cancers [92,93], cholangiocarcinoma [94], hepatocellular and small-cell lung carcinoma [39]. Regarding pediatric cancers, CDH6 overexpression was observed in osteosarcoma and was associated with the overall survival and prognosis of osteosarcoma patients [95], where CDH6 expression was regulated by miR-223-3p. CDH6 seems to be strongly expressed in aggressive thyroid carcinomas [96] and nasopharyngeal carcinoma [97]. In addition, RNA-seq data and tissue immunohistochemistry confirmed the restricted normal tissue distribution of CDH6 and the preferential expression in renal and ovarian cancers [98]. To note that CDH6 overexpression seems to be related to cancers originating from developmentally related mullerian, renal and thyroid lineages. Transcription factor Paired Box 8 (PAX8), common to these three lineages, seems to regulate the expression of CDH6 [99]. Interestingly, in some ovarian cancers, overexpression of CDH17 has been reported, suggesting that both cadherins might coexist in some cancer tissues. A recent report has investigated the association of cadherins, including CDH6 and CDH17, with stem-cell-related transcription factors in triple negative breast cancer (TNBC) [100]. There was a significant percentage of TNBC samples from TCGA datasets with alterations in CDH17 (20%) and CDH6 (14%). In addition, a clear concurrence of stem-cell-related transcription factors (Forkhead Box M1 (FOXM1), SNAI1, SRY Box 9 (SOX9), Minichromosome Maintenance Complex Component 2 (MCM2), among others) expression with alterations in CDH6 and CDH17 was observed. The authors propose that the loss of E-cadherin is compensated by the upregulation of several other cadherins, including CDH6 and CDH17, as well as an increased expression of transcription factors such as Snail, SOX9 or FOXM1.

Regarding signaling, CDH6 was described as a TGF-β target gene in thyroid cancer cell lines to promote a mesenchymal phenotype [101]. However, these results were conflicting with the functional role of CDH6 in epithelial morphogenesis. Furthermore, the transcription factor RUNX2 was proposed to be involved in the regulation of CDH6 in these cell lines [101], conflicting with the reports of PAX8 implication. In addition, CDH6 was reported to interact with autophagy-related proteins GABA Type A Receptor-Associated Protein (GABARAP), BCL2 Interacting Protein 3 (BNIP3) and BNIP3L to restrict autophagy and to promote the reorganization of the mitochondrial network in thyroid cancer cells [102] (Figure 1). The interaction with the autophagic proteins is mediated by short motifs called LC3-interacting (LIR) domains. These LIR domains seem to be present in other cadherins such as VE-cadherin, but not in the 7D cadherins (CDH17 and CDH16), probably due to the short cytoplasmic domain. Although we might assume a relationship between cadherin re-expression in advanced neoplasias as being related to epithelial plasticity and reacquisition of a more epithelial phenotype, the link between EMT and autophagy is unclear. It has been described that autophagy improves viability and survival by increasing anoikis resistance and immune surveillance escape.

## 4. Phylogenetic Analysis of the RGD Motif in Cadherins

**Figure 2:**
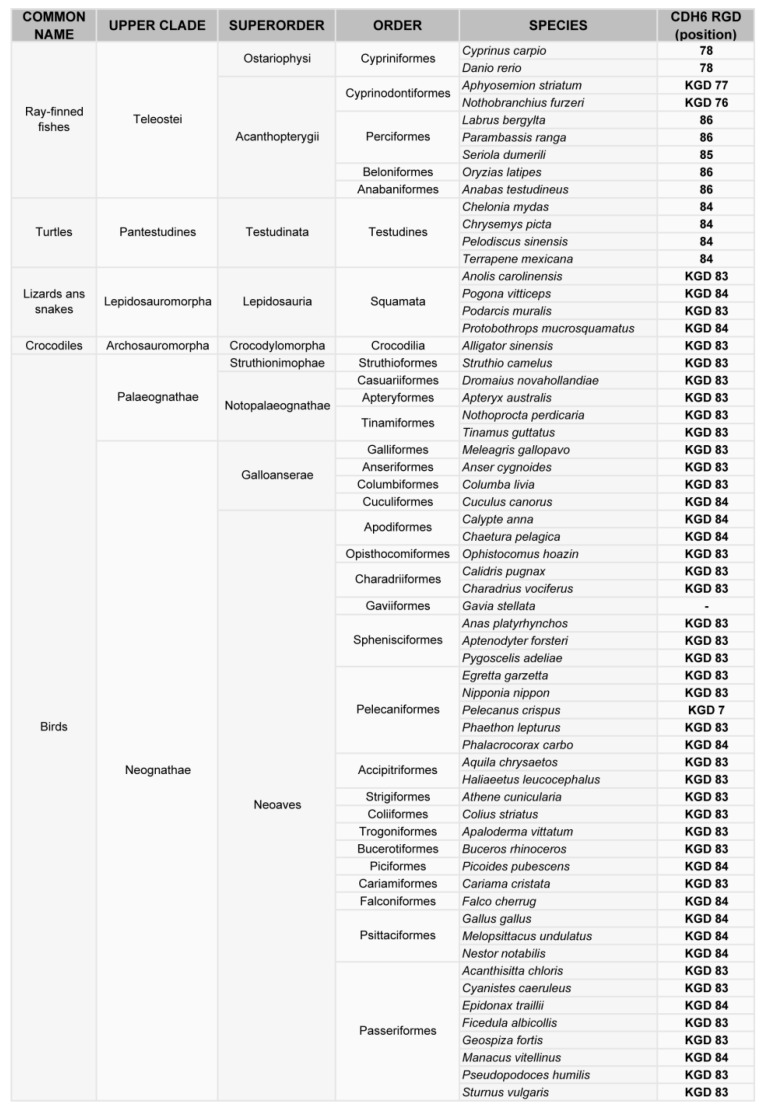
Phylogenetic analysis of the RGD motif in CDH6. Classification of vertebrates (excluding mammals) where the CDH6 gene has been sequenced, indicating the position of the RGD or KGD motif in the sequence.

**Figure 3:**
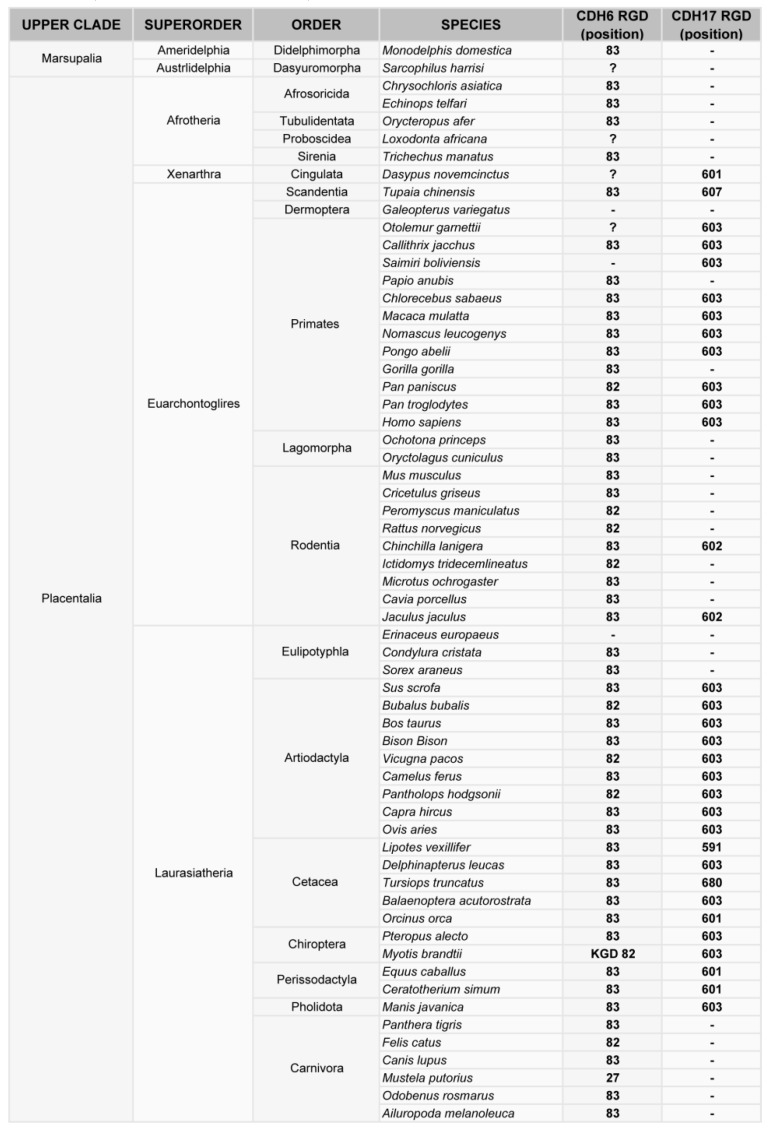
Phylogenetic analysis of the RGD motif in CDH6 and CDH17 in mammals. Classification of mammal species where CDH17 and/or CDH6 have been sequenced, showing the position of the RGD motifs in such cadherins.

Phylogenetic analysis of cadherin sequences reveals the evolutionary history of the RGD motif in cadherins. The RGD motif in CDH6 appears very early in evolution, as most fish (80% of analyzed sequences) have this motif around position 86 in the CDH6 amino acid sequence. This motif is highly conserved in mammals, fishes and turtles (Figure 2, Figure 3). However, the diapsida (lizards, crocodiles and birds) present a mutation that changes the RGD motif into a KGD motif (Figure 3). In contrast, RGD motifs in CDH17 and VE-Cadherin are more recent in evolution. The RGD motif in CDH17 is exclusively present in placental mammals (57%, position 603) (Figure 2). To note the lack of RGD motif in the CDH17 sequence of carnivores, rodents (mouse, rats) and lagomorphs (rabbits). Something similar happens for the VE-cadherin RGD motifs, whereas RGD in VE-cadherin sequence is found in placental mammals (43%) and birds (64%) with similar exceptions [31]. However, the position of the RGD motif in VE-cadherin shows a parallel development, as it is present in cadherin EC domain 2 in mammals but in domain 3 in birds. In fact, the RGD motif in VE-cadherin has appeared several times in evolution, as placental mammals show this motif in different positions: 83, 191 and 236 in the amino acid sequence. In addition, primates show a second RGD motif in position 299 (in the cadherin domain 3) [31].

The physiological role of RGD motifs in cadherins has been studied only in CDH6. This RGD cadherin is expressed in platelets and plays a key role in coagulation through the activation of αIIbβ3 integrin [103]. Such an important function might explain the early origin of this motif. The KGD mutation found in many reptiles and birds probably has no deleterious effect, as this motif is likely recognized by αIIbβ3 integrin [104,105]. The role of RGD motifs in other cadherins has not been studied in physiological conditions. However, since CDH17 promotes cell adhesion and proliferation through activation of α2β1 integrin [20], we tentatively propose that the CDH17 RGD motif can promote enterocyte cell–cell adhesion and, particularly, cell proliferation. In fact, intestinal epithelium is the human tissue with the highest proliferation and replacement rate. Regarding the VE-cadherin RGD motif, this cadherin promotes cell adhesion and proliferation, but also increases their migratory/invasive behavior in tumor cells. VE-cadherin is mainly expressed in endothelial cells, where its RGD motif could promote cell migration through activation of α2β1 integrin. The α2β1-triggered migration of endothelial cells is known to be of key relevance in angiogenesis [106]. Therefore, VE-cadherin RGD motif may promote angiogenesis and vascular development in homeotherm animals (mammals and birds).

## 5. Cadherin Interaction with Integrins: Activation of the Integrin Signaling Pathway by Cadherin RGD Motifs

Integrins are heterodimeric receptors formed by different combinations of 18 α subunits and 8 β subunits in mammalians [107]. Integrin bi-directional signaling plays a key role in multiple cellular functions including proliferation, adhesion, migration and invasion [107]. The integrin repertoire fluctuates widely between different cell lines impacting cancer development and progression. In general, these alterations are poorly characterized in solid tumors due to the large number of potential integrin heterodimers. Initially, metastasis-associated integrins were mainly limited to the canonical RGD receptor integrins, such as αvβ3, αvβ5, α4β1 and α5β1 (see [108,109] for reviews). They were preferentially associated with cancer metastasis, angiogenesis and therapy rather than α2β1 integrin. Only recently, α2β1 integrin has received more attention in metastasis research due to the accumulation of evidence that supports a critical role for this integrin in cancer metastasis, including its capacity to bind RGD-containing ligands (see [110] for a review).

**Figure 4:**
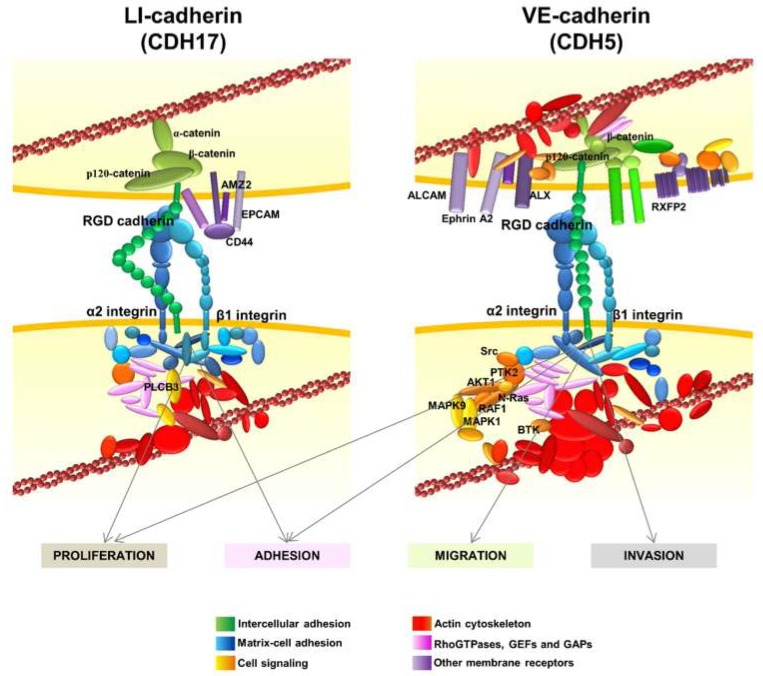
RGD cadherin interactome and integrin signaling in cancer cells. Diagram showing the proteins that specifically bind to CDH17 (left) or VE-cadherin (right) by proteomic analysis and data mining. The most relevant proteins are indicated. Interaction with the RGD cadherins leads to the activation of α2β1 integrin signaling, which promotes proliferation and adhesion in cancer cells, and also migration and invasion in breast cancer and melanoma cells expressing VE-cadherin. Adapted from [17,31].

In general, the studies of the interplay between cadherins and integrins have been limited to E-cadherin and N-cadherin. A summary of the interactions between E-cadherin and N-cadherin with different integrins was published by Weber et al. [111]. However, interactions of E-cadherin with different integrins always occurs in an indirect way, forming ternary complexes with other proteins such as insulin-like growth factor 1 receptor [112] or Cell Division Cycle 42 (CDC42) [113]. No direct binding motifs to integrins have been described for E- or N-cadherin to the best of our knowledge. The first direct binding motif to integrins found in cadherins was reported for CDH17 binding to α2β1 integrin through the RGD motif in metastatic colorectal cancer cells [20]. Then, we described the interaction of CDH17 and VE-cadherin with α2β1 integrin in different cellular contexts and cancer models (Figure 4) [17,20,31]. This interaction was facilitated and promoted by the presence of one or two RGD motifs in CDH17 and CDH5, respectively [20,31]. At the same time, Siret et al. [114] described the association of α2β1 integrin with both E-cadherin and N-cadherin to regulate melanoma cell invasion. Both cadherins led to different patterns of co-localization and functional activity. Whereas the interaction with N-cadherin promoted invasion and migration towards type I collagen, interaction with E-cadherin promoted cell–cell adhesion, suggesting differential cell responses according to the associated cadherin. However, no other cadherins were investigated in this report. It is unclear how the interaction between E- and N-cadherin and α2β1 integrin and other integrins takes place as no RGD motifs are present in these two cadherins. It might be possible that heterophylic interactions between different cadherins are formed or multiple cadherins coexist in the same cells. It seems evident that future interaction studies between cadherins and integrins should not be restricted to the E- and N-cadherins. In any case, interactions between cadherins and integrins have been widely observed and they can modulate a large range of cellular functions, including cytoskeletal organization, cell polarity, cell proliferation and cell adhesion [111]. Therefore, it is critical to understand the molecular mechanisms that drive this interaction and to clarify if there are other molecular motifs involved beyond the RGD motif.

## 6. Other Interactions of RGD Cadherins Involved in Cell Signaling

In addition to the α2β1 integrin, the interactome network of CDH17 revealed several other protein clusters (Figure 4) [17]. Many proteins from the actin cytoskeleton including the main regulators of actin polymerization, the RhoGTPases and their regulators, were identified. Another group of proteins associated with CD44, such as epithelial cell adhesion molecule (EPCAM), galectin 3 (Gal3) and Archaelysin Family Metallopeptidase 2 (AMZ2), were identified in the CDH17 network. This is relevant as CD44 and EPCAM are markers associated with stemness and differentiation. Galectin 3, which was found to cluster with CDH17 in pancreatic ductal adenocarcinoma [49], is involved in the canonical Wnt/β-catenin signaling pathway and in the accumulation of nuclear β-catenin in colorectal cancer [115]. This effect would agree with the reported activation of Wnt signaling when CDH17 is up-regulated in hepatocellular carcinoma [57]. Laminin-interacting α6β4 integrin was also present in the CDH17 network, although a direct interaction was not demonstrated and no role for α6β4 integrin in the pro-metastatic proteins of CDH17 was detected. Thrombospondin 2 and sindecan 1 were also identified as partners of the CDH17 complex as well as α-, β-, and p120-catenins, suggesting that CDH17 can bind these proteins even with a short cytoplasmic tail, without homology to those of classical cadherins.

The interactome of VE-cadherin was quite similar to the CDH17 network, including matrix–cell adhesion proteins, actin cytoskeleton and Rho-GTPase associated proteins (Figure 4). As expected, catenins and other cell–cell adhesion proteins were also identified. Despite the overall functional similarity between protein clusters in CDH17 and VE-Cadherin networks; the exact correspondence between proteins was low between both interactomes, probably due to the different cell context in which the cadherins were expressed: CDH17 in colorectal cancer and VE-cadherin in melanoma and breast cancer. Several membrane receptors were identified as part of the VE-cadherin network such as activated leukocyte cell adhesion molecule (ALCAM), ALX homeobox 1 (ALX), EPHA2 and G-protein coupled receptors. High ALCAM expression at the protein level usually correlates with a poor prognosis in melanoma, breast and colorectal cancer, among others [116]. ALCAM expression correlates with melanoma progression, as it is much more abundant in late stages [117]. Note the presence of EPHA2 that was regulated by CDH5 for vasculogenic mimicry in melanoma cells [87]. However, the main difference with CDH17 clusters was the identification in VE-cadherin network of many signaling proteins such as Src kinases, Ras, Phosphoinositide-3-Kinase (PI3K) and Mitogen-Activated Protein Kinase (MAPK) pathway proteins and several phosphatases. Therefore, whereas CDH17 triggers the activation of MAP kinases and Focal Adhesion Kinase (FAK), VE-cadherin can activate Src and PI3K pathways in addition to MAP kinases. These results might explain the different effects of both RGD cadherins in the context of cancer cells: whereas CDH17 promotes cell adhesion and proliferation, VE-cadherin increases cell migration and invasion in addition to adhesion and proliferation [20,31]. The association with other important epithelial/stemnicity markers, such as CD44 and EPCAM with CDH17, and ALCAM and AXL with VE-cadherin, remains to be studied in depth. Potential modulation of functions of these proteins by RGD cadherins could be of key importance in cancer progression. The network of CDH6 remains to be studied in detail.

## 7. Targeting Cadherins: Implications in Therapy

An overview of the reported participation of RGD cadherins in cancer progression and metastasis indicate a wide number of cancer types and subtypes as indicated in the previous sections. This list is probably not exhaustive and is likely to expand in the next years, making these cadherins interesting targets for therapy.

Since E-cadherin expression declines during metastatic progression, it has not typically been considered as a direct therapeutic objective but used as an indirect target for epigenetic regulation. Alternatively, since N-cadherin has been implicated in cancer metastasis endowing tumor cells with enhanced migratory and invasive capacity, a variety of N-cadherin antagonists have been developed and tested. A monoclonal antibody called GC-4, which binds to the EC1 domain of N-cadherin is able to block N-cadherin-mediated adhesion and might be relevant to limit metastatic dissemination [13]. GC-4 blocking ability also increased tumor cell sensitivity to imatinib, to overcome tyrosine kinase inhibitor resistance [118]. Another additional therapeutic strategy made use of a stable cyclic peptide called ADH-1 that harbors a HAV motif used for lateral clustering of cadherins [119]. As Fibroblast Growth Factor Receptor 1 (FGFR1) contains a HAV motif, it was assumed that these peptides might also inhibit FGFR signaling. The efficacy of this peptide has been demonstrated in a number of models of pancreatic, breast, colon, ovarian and lung cancer (see [120] for a review). Its efficacy remains to be demonstrated in clinical studies.

**Figure 5:**
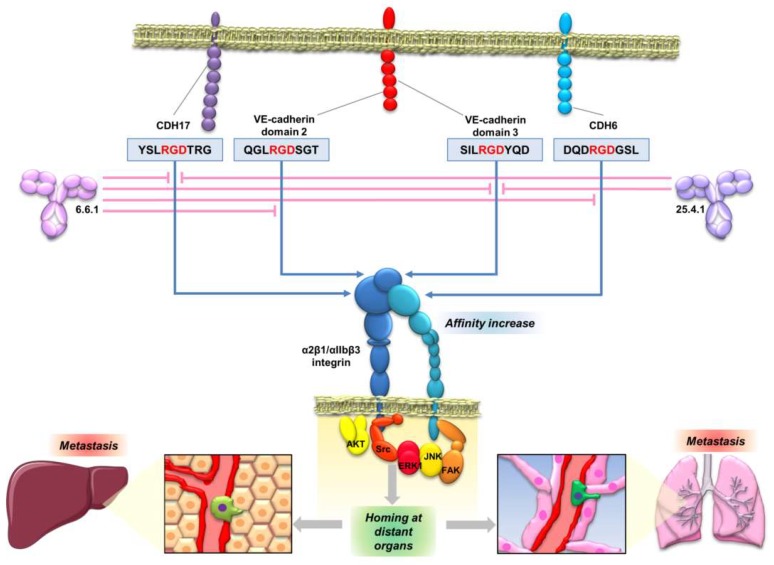
Anti-cadherin RGD antibodies as a potential therapeutic strategy against metastasis. Schematic representation of the activity of two anti-cadherin RGD monoclonal antibodies, showing their specificities against RGD motifs in cadherins. The antibodies can inhibit the interaction of the RGD motifs with the integrins, blocking their activation, signaling and consequently, the metastatic behavior of cancer cells.

The interaction of CDH17 and other cadherins with α2β1 integrin mediated by an RGD motif enables the development of new therapeutic opportunities based on the inhibition of the cadherin RGD binding. Peptides containing the cadherin RGD motif were used to prepare specific monoclonal antibodies. These monoclonal antibodies blocked the cadherin-mediated β1 integrin activation in colorectal, pancreatic, breast and melanoma cancer cell lines [121] (Figure 5). Therefore, the monoclonal antibodies, particularly antibody 6.6.1, blocked the integrin signaling pathway activation of FAK in colorectal cancer, of JNK and ERK kinases, in colorectal and pancreatic cancers, and of JNK, ERK, Src and AKT in melanoma and breast cancers (Figure 4 and Figure 5). These RGD-specific monoclonal antibodies were highly effective in prolonging mice survival in mouse models of liver metastasis (colorectal cancer and melanoma) and lung metastasis (melanoma). Humanized versions of 6.6.1 are being prepared for preclinical studies (unpublished data). Once the effectiveness of these cadherin RGD-specific monoclonal antibodies in cancer models overexpressing CDH17 and VE-cadherin has been demonstrated, the next step should be to investigate the efficacy of these antibodies in other cancers positive for high CDH6 expression, such as ovarian, renal or thyroid cancers.

CDH6 has also been identified as a target for biotherapeutics development. The recent discovery and optimization of an antibody-drug conjugate (ADC) directed against this protein has yielded effective results and provided beneficial effects in the treatment of ovarian and renal carcinomas [98]. However, these antibodies used for CDH6 ADC-targeted treatment were not RGD-specific. They bound to a conformational epitope situated in the EC5 domain, working as carriers to vectorise the drug to the cancer cells. Indeed, a naked version of this antibody lacked antitumor efficacy [98].

Targeting of cadherins for cancer treatment raises some questions about the potential complications of targeting proteins with multiple relevant physiological roles. In this regard, although GC-4 antibody or ADH-1 peptide affect the N-cadherin-mediated cell adhesion, ADH-1 seems to be well tolerated in humans [122]. Regarding the anti-cadherin RGD monoclonal antibodies, they have no effect on the main cadherin function: the homotypic cell–cell adhesion, as it is RGD-independent [20]. Moreover, the treatment should affect only cells that simultaneously express RGD cadherins together with α2β1 or αIIbβ3 integrins. Indeed, mouse models where 6.6.1 antibody was used as a treatment for metastasis did not show any side effects [121]. It is noteworthy that the expression levels of cadherins are quite lower in healthy tissues compared with several tumors, where such cadherins are prominently overexpressed or “de novo” expressed, suggesting that most of the targeting will occur in cancer cells. However, it remains to be demonstrated in further clinical trials.

## 8. Final Remarks and Outlook

The three cadherins discussed here share some common features, such as the presence of RGD motifs in the extracellular domains. This RGD motif in CDH17 and VE-cadherin enables the activation of α2β1 integrin in different cancers and the activation of αIIbβ3 integrin in CDH6-expressing tumors (manuscript in preparation). In addition, they are usually re-expressed in advanced metastatic stages of cancer associated to a more epithelial phenotype. They are usually associated with cellular plasticity, including the mesenchymal-to-epithelial transition (MET) and the gain of stem-cell-like properties in metastatic cells, as revealed by the increased expression of CD44, EPCAM and ALCAM, among other markers. The upregulation in late-stage tumors in association with key integrins promotes different pro-metastatic properties in different cancers that support the use of these cadherins as therapeutic targets.

Still, some critical questions remain to be answered such as the potential correlation between the expression of N-cadherin and the RGD cadherins. The potential interaction between N-cadherin and other cadherins has never been explored to the best of our knowledge. The simultaneous co-expression of several cadherins and potential synergies in some tumors deserves further exploration. The involvement of other integrins, such as αIIbβ3, in the pro-metastatic mechanisms of CDH6 is currently the subject of research. The presence of soluble fragments of CDH17 and VE-cadherin released by cancer cells may facilitate the signaling capacity with integrins and the formation of trans-interactions between cadherins. Soluble fragments may compete with homotypic interactions to reduce the intercellular adhesion. The proteases involved in this shedding still need to be identified for CDH17. It is also unclear if CDH6 ectodomains are released as soluble fragments to participate in cell signaling. The different position of the RGD motifs in different extracellular domains might be related with a different signaling capacity. Undoubtedly, further exploration of N-cadherin and RGD cadherins’ role in cancer metastasis promotion is needed to develop new and more efficient therapeutic strategies.
